# Prognostic value of the modified systemic inflammation score in non-small-cell lung cancer with brain metastasis

**DOI:** 10.1186/s12935-022-02704-w

**Published:** 2022-10-14

**Authors:** Feng Wang, Limin Chen, Zhao Wang, Qiuyan Xu, He Huang, Hairong Wang, Xi Li, Mingjie Yu, Jiangen Chen, Fuhua Lin, Zhenghe Chen, Xiangheng Zhang, Qunying Yang, Yonggao Mou, Chengcheng Guo

**Affiliations:** 1 Department of Neurosurgery, Sun Yat-Sen University Cancer Center, The State Key Laboratory of Oncology in South China, Collaborative Innovation Center for Cancer Medicine, 651 Dongfeng Road East, Guangzhou, 510060 China; 2 Department of Oncology, Affiliated Hospital of Binzhou Medical College, 346 Guanhai Road, Binzhou, 264003 China; 3 Department of Medical Oncology, Sun Yat-Sen University Cancer Center, The State Key Laboratory of Oncology in South China, Collaborative Innovation Center for Cancer Medicine, 651 Dongfeng Road East, Guangzhou, 510060 China

**Keywords:** Non-small-cell lung cancer, Brain metastasis, Systemic inflammation score, Neutrophil–lymphocyte ratio, Albumin, Prognosis

## Abstract

**Background:**

Brain metastases (BM) from non-small-cell lung cancer (NSCLC) is the most common brain malignancy. Systemic inflammation biomarkers have recently been evaluated as prognosis indicators in several tumors. The combination of these markers has not been evaluated in NSCLC with BM yet. Here, we explored the predictive value of pretreatment inflammatory biomarkers and established a novel, clinically applicable prognostic index for NSCLC patients with BM.

**Methods:**

A retrospective investigation of 951 NSCLC patients newly diagnosed with BM at Sun Yat-sen University Cancer Center was conducted. We randomly divided patients into a training cohort (n = 674) or validation cohort (n = 277). Receiver operating characteristic (ROC) curve analysis was carried out to obtain the optimal cut-off values of pretreatment systemic inflammatory indexes. The associations between serum biomarkers and overall survival (OS) were analyzed by Kaplan–Meier curves and Cox proportional models. The resulting prediction model has been externally verified through the validation cohort.

**Results:**

The optimal cut-off value of the neutrophil–lymphocyte ratio (NLR) in predicting OS was 4.71, while the clinical standard of 40 mg/L was chosen as the optimal cut-off value of albumin. Univariate and multivariate analyses revealed that patients receiving local treatment, chemotherapy, a NLR < 4.71 and albumin ≥ 40 mg/l independently predicted improved survival. We combined the two inflammatory indexes (NLR and albumin level) to establish the modified systemic inflammation score (mSIS) which divides patients into low risk, medium risk or high-risk groups. The 1-year OS rates of three groups were 59.7%, 40.5% and 29.4%, respectively in the training cohort. The same result was verified in the validation cohort with the 1-year OS rates 69.7%, 47.0% and 7.7%, respectively. The mSIS exhibited better discrimination power than the American Joint Committee on Cancer’s (AJCC) 7th T + N staging system in the training cohort (Harrell’s concordance index (C-index): 0.744 vs 0.502, P < 0.05), and the discrimination was also superior to that of AJCC’s 7th T + N staging system in the validation cohort (C-index: 0.724 vs 0.527, P < 0.05). The 1-year and 2-year OS rates of the AUC also exhibited superior survival predictive ability to that of the AJCC’s 7th T + N staging system in NSCLC patients with BM.

**Conclusion:**

The pretreatment mSIS may be an independent prognostic factor for OS in NSCLC patients with BM and warrants further research.

## Introduction

Lung cancer is one of the malignant tumors with the highest morbidity and mortality worldwide [1]. Currently, non-small-cell lung cancer (NSCLC) accounts for more than 80% of all lung cancers, with 30–50% of patients developing brain metastases (BM) [2, 3]. About 10% of NSCLC patients have synchronous BM at their initial diagnosis, while 40–50% of patients develop metachronous BM during the course of the disease [4–6]. With the rapid development of immunotherapy, targeted therapy and other treatment methods, the survival time of lung cancer patients has gradually prolonged, but the number of patients with BM has gradually increased too [7]. Due to the blood–brain barrier, patients with BM are usually less sensitive to drug therapy and have a poor overall prognosis [8, 9]. It is of great significance and necessity to explore new survival predictive markers that can be used for risk stratification and clinical decision-making for NSCLC patients with BM.

In previous studies, the prognostic factors for evaluating lung cancer were mainly focused on genetic testing, biological indicators, tumor size, clinical stage, and pathologic types [10]. Therefore, simple and non-invasive factors that preoperatively predict NSCLC with BM would be of great utility and value. The systemic inflammation and immune status of the body has been shown to play a considerable role in the development of cancer [11, 12]. Moreover, inflammation may cause the tumor microenvironment to promote cancer progression [13]. Our group previously combined the neutrophil–lymphocyte ratio (NLR) and albumin as two inflammatory markers to establish the modified systemic inflammation score (mSIS), which has been preliminarily confirmed as an independent prognostic factor for overall survival (OS) in extranodal NK/T cell Lymphoma (ENKTL) [14].

However, to the best of our knowledge, there is no report on mSIS for NSCLC patients with BM. Here, this study explores the prognostic value of the pretreatment serum-based inflammatory biomarkers to establish a scoring system for NSCLC patients with BM to improve physician treatment decision making.

## Patients and methods

### Ethical statement

This study was approved by the Medical Ethics Committees of Sun Yat-sen University Cancer Center (approval number B2022-290–01). Written informed consent was obtained from each participant to use data from the hospital’s database. This study was conducted in accordance with the World Medical Association’s Declaration of Helsinki, and the relevant guidelines and regulations.

### Study population

A database consisting of 951 patients diagnosed with primary NSCLC with BM was retrospectively surveyed at Sun Yat-sen University Cancer Center from January 1990 to December 2010. Eligible patients were histologically confirmed based on the 2008 World Health Organization (WHO) criteria. BM was confirmed by surgical pathology or clinical diagnosis (such as clinical symptoms, brain computed tomography or nuclear magnetic resonance). The eligibility criteria were: patients with complete follow-up data and clinical information, patients who had not received any anticancer therapies at the time of the initial diagnosis, and patients who had no preceding malignant or secondary tumors. The exclusion criteria were: patients with acute chronic active inflammatory or infectious diseases; patients with incomplete/inaccurate medical records.

### Data collection

The baseline characteristics, treatment process, and outcomes of all patients were collected via medical records. The TNM stages of patients were determined based on the 7th American Joint Committee on Cancer (AJCC) edition staging for NSCLC. For all patients, fiberoptic bronchoscopy, contrast-enhanced thoracic and upper abdominal computed tomography (CT) scanning, and contrast-enhanced cerebral magnetic resonance imaging (MRI) were performed before treatment. Because of BM, a comparative brain MRI was performed every 3 months to evaluate the cerebral disease. All patients were evaluated for blood cell counts and albumin level before treatment. Follow-up was performed from the date of diagnosis. Patients were generally followed-up every 3 months in the first year, every 6 months in the second and third year, and annually thereafter. On the final follow-up date, clinical visits were made by direct communication with the patients or their family members. OS was defined as the time between the date of diagnosis and the date of death or the final follow-up period.

### Statistical analysis

We applied the Chi-square test or Fisher’s exact test to evaluate varying baseline and clinicopathological parameters according to the type of data. The optimal cut-off value of the NLR was determined by the receiver-operating characteristic (ROC) curve. The optimal cut-off value of albumin is the standard clinical value. Univariate Cox regression analyses and multivariate proportional hazards regression models were carried out to identify independent prognostic factors. Survival analysis among groups was assessed using the Kaplan–Meier method and log-rank tests. Harrell’s concordance index (C-index) and ROC curves were calculated to evaluate the predictive efficacy of the models using R version 4.0.2 via the survival and design packages. Other statistical analyses were performed using SPSS version 25.0 (IBM Corp, Armonk, NY, United States). All reported P-values were two-sided, and P < 0.05 was considered statistically significant. CIs were calculated at the 95% level.

## Results

The clinical features of 951 patients, including the training cohort (n = 674) and the validation cohort (n = 277) are summarized in Table 1. The median age of all patients at diagnosis was 68 years old (range, 23–81 years). There were 644 males (67.7%) and 307 females (32.3%). Most patients (678/951, 71.3%) had a history of smoking. 77.2% (734/951) of patients received systemic chemotherapy, while 57.1% (543/951) of patients received local treatments including surgery, whole-brain radiation therapy (WBRT) and stereotactic radiosurgery (SRS). The median size of the largest BM lesion was 1.97 cm (0.2–6.0 cm). In addition, 33.8% (321/951) of patients had different levels of symptomatic BM. 76.6% (728 patients) had synchronous metastases and 23.4% (223 patients) had metachronous metastasis. Patients in the two cohorts exhibited no significant differences regarding most clinical features.

**Table 1:** Baseline characteristics of patients

Characteristics	Training cohort			Validation cohort	
	No. of patients		%	No. of patients	**%**
Age(year)					
< 65	200		29.7	91	32.9
≥ 65	474		70.3	186	67.1
Gender					
Male	462		68.5	182	65.7
Female	212		31.5	95	34.3
Smoking					
Smoker	490		72.7	188	67.9
Never-smoker	184		27.3	89	32.1
PS					
0–1	211		31.3	3	1.1
≥ 2	463		68.7	274	98.9
T stage					
1	97		14.1	23	8.3
2	285		42.3	135	48.7
3	147		21.8	59	21.3
4	145		21.5	60	21.7
N stage					
0	92		13.6	31	11.2
1	133		19.7	60	21.7
2	228		33.8	97	35
3	221		32.8	89	32.1
Synchronous BM					
Yes	518		76.9	210	75.8
No	156		23.1	67	24.2
Number of BM lesion					
1	261	38.7		107	38.6
≥ 2	413	61.3		170	61.4
Maximum diameter of BM lesion					
< 2 cm	360	53.4		174	62.8
≥ 2 cm	314	46.6		103	37.2
Extracranial metastases					
Yes	259	38.4		110	39.7
No	415	61.6		167	60.3
Cerebral symptoms					
Yes	234	34.7		87	31.4
No	440	65.3		190	68.6
Receiving local treatment (surgery/wbrt/srs)					
Yes	412	61.1		131	47.3
No	262	38.9		146	52.7
Receiving chemotherapy					
Yes	517	76.7		217	78.3
No	157	23.3		60	21.7

*PS*,Performance Status; *BM*, Brain metastases; *WBRT*, whole-brain radiation therapy; *SRS*, stereotactic radiosurgery

We analyzed the cut-off value of the NLR based on the training cohort. According to the ROC curve, the area under the curve (AUC) of the NLR was 0.608 (95% CI 0.560–0.657; P < 0.001) based on the cut-off value of 4.71. The optimal cut-off value for albumin was 40 mg/L. The NLR-low group (78.8%, 531/674) had a higher OS than the NLR-high group (21.2%, 143/674; P < 0.001). The 1-year OS rates of the NLR-low group and the NLR-high group were 49.1% and 31.5% respectively. OS was significantly poorer in the albumin-low group (58.6%, 395/674) than in the albumin-high group (41.4%, 279/674; P < 0.001), while the 1-year OS rates in the albumin-low group and albumin-high group was 39.0% and 54.5%, respectively. Table 2 shows the results of the univariate analysis of clinical variables that were considered predictors of OS. The following clinical factors significantly predicted survival in the univariate analysis: smoking history, maximum diameter of BM lesions, local treatment, chemotherapy, NLR and albumin level. The selected variables with their associated hazard ratios (HRs) are presented in Table 3.

**Table 2:** Univariate analyses of potential prognostic factors for OS using the training cohort

Characteristics	HR (95% CI)	*P* value
Age(year)		
< 65	1	
≥ 65	1.010(0.841–1.213)	0.913
Gender		
Male	1	
Female	1.038(0.868–1.242)	0.68
Smoking		
Smoker	1	
Never-smoker	0.811(0.669–0.984)	0.033
PS		
0–1	1	
≥ 2	1.169(0.980–1.395)	0.083
Synchronous BM		
Yes	1	
No	0.919(0.752–1.123)	0.409
Maximum diameter of BM lesions		
< 2 cm	1	
≥ 2 cm	1.235(1.046–1.459)	0.013
Receiving local treatment (Surgery/WBRT/SRS)		
Yes	1	
No	2.199(1.852–2.610)	< 0.001
Receiving chemotherapy		
yes	1	
no	1.897(1.573–2.289)	< 0.001
NLR		
≥ 4.71	1	
< 4.71	0.568(0.468–0.689)	< 0.001
Albumin		
< 40 mg/l	1	
≥ 40 mg/l	0.523(0.438–0.625)	< 0.001

*PS*, Performance Status; *BM*, Brain metastases; *WBRT*, whole-brain radiation therapy; *SRS*, stereotactic radiosurgery; *NLR*, neutrophil–lymphocyte ratio

**Table 3:** Selected factors according to the Cox PHs regression model based on the training and validation cohorts

Characteristics	Training set		Validation set	
	HR (95% CI)	*P* value	HR (95% CI)	*P* value
Receiving local treatment (Surgery/WBRT/SRS)				
Yes	1		1	
No	1.769(1.471–2.127)	< 0.001	1.806(1.504–2.169)	< 0.001
Receiving chemotherapy				
Yes	1		1	
No	1.313(1.082–1.618)	0.006	1.318(1.078–1.612)	0.007
NLR				
≥ 4.71	1		1	
< 4.71	0.630(0.517–0.767)	< 0.001	0.634(0.521–0.772)	< 0.001
Albumin				
< 40 mg/l	1		1	
≥ 40 mg/l	0.617(0.514–0.741)	< 0.001	0.610(0.508–0.732)	< 0.001

*WBRT*, whole-brain radiation therapy; *SRS*, stereotactic radiosurgery; *NLR*, neutrophil–lymphocyte ratio

Although our group has demonstrated good predictive value in ENKTL with the combination of NLR and albumin level (the mSIS), whether it can predict the prognosis of NSCLC patients with BM remains unclear. Here, we first demonstrate the prognostic value of the mSIS on the prognosis of NSCLC patients with BM.

**Fig. 1:**
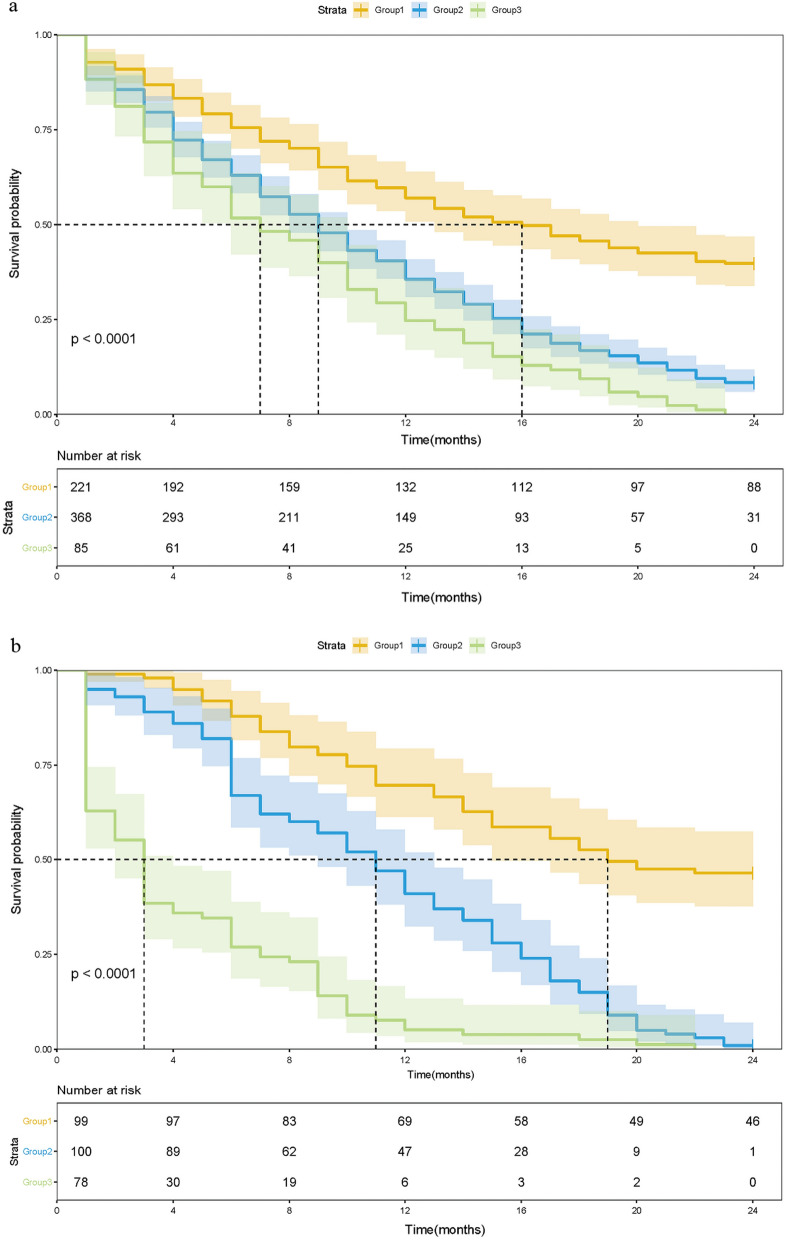
Kaplan–Meier survival analysis for OS according to the mSIS in the training cohort (**a**) and validation cohort (**b**)

In the training cohort, 674 patients were divided into three groups: Group 1 (mSIS score = 0, low risk), patients with NLR < 4.71 and albumin ≥ 40 mg/L (221 cases, 32.8%); Group 2 (mSIS score = 1, medium risk), patients with NLR ≥ 4.71 and albumin ≥ 40 mg/L (310 cases, 46.0%) or patients with NLR < 4.71 and albumin < 40 mg/L (58 cases, 8.6%); and Group 3 (mSIS score = 2, high risk), patients with NLR ≥ 4.71 and albumin < 40 mg/L (85 cases, 12.6%). The 1-year OS rates of these three groups were 59.7%, 40.5% and 29.4% respectively (P < 0.001, Fig. 1a). In the validation cohort, 277 patients were also divided into three groups: Group 1 (mSIS score = 0, 99 cases, 35.7%); Group 2 (mSIS score = 1, 100 cases, 36.1%); and Group 3 (mSIS score = 2, 78 cases, 28.1%). The 1-year OS rates of these three groups were 69.7%, 47.0% and 7.7%, respectively (P < 0.001, Fig. 1b).

We compared the discrimination of the mSIS with the 7th AJCC’s T + N classification in the training cohort. The C-index was 0.744 (95% CIs 0.707–0.781), which was superior to the 7th AJCC’s T + N classification (0.502, 95% CIs 0.465–0.539, P < 0.05). Discrimination was also enhanced compared with the 7th AJCC’s T + N classification in regard to the validation cohort (C-index 0.724, 95% CIs: 0.667–0.781 vs 0.527, 95% CIs: 0.471–0.583, P < 0.05).

**Fig. 2:**
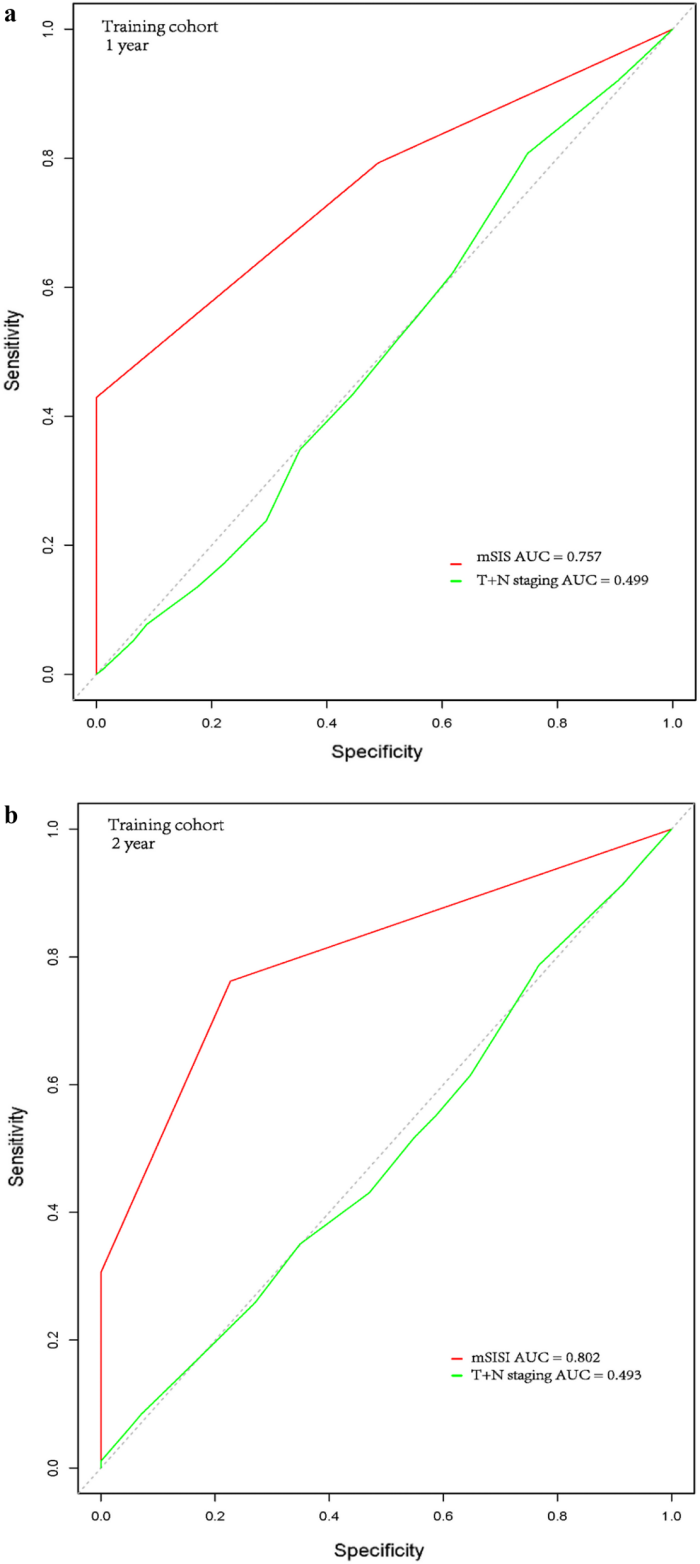

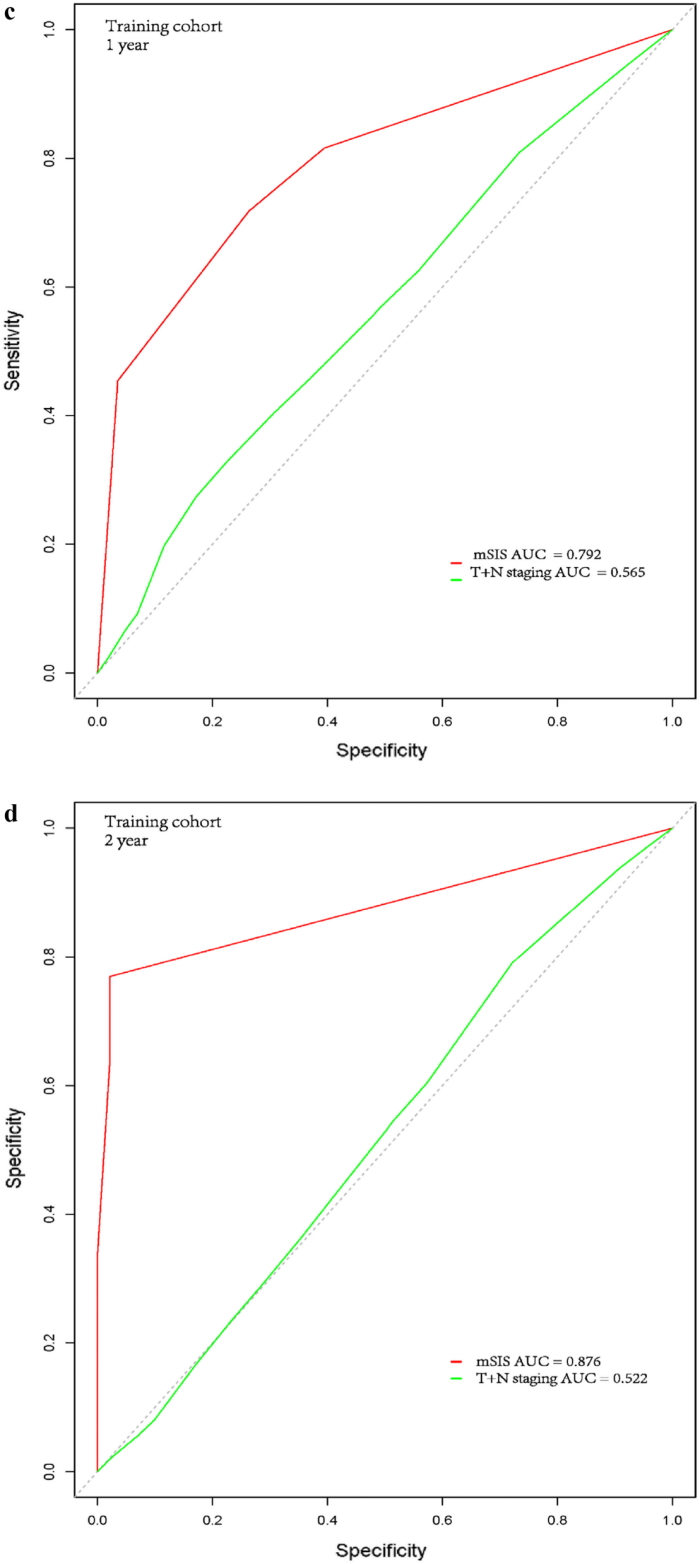
The ROC courves of mSIS for predicting the 1-year and 2-year OS rates in the training cohort (**a**,**b**) and validation cohort (**c**,**d**)

The AUC for the 1-year and 2-year OS rates regarding the prediction ability of the two data cohorts were compared (Fig. 2a–d). For the training cohort, the AUCs of the mSIS in predicting the 1-year and 2-year OS rates were 0.757 and 0.802 respectively, whereas, the AUCs of the 7th AJCC’s T + N classification in predicting the 1- and 2-year OS rates were 0.499 and 0.493, respectively. Regarding the validation cohort, the AUCs of mSIS in predicting the 1- and 2-year OS rates were 0.792 and 0.876, respectively, whereas, the AUCs of the 7th AJCC’s T + N classification in predicting the 1- and 2-year OS rates were 0.565 and 0.522, respectively. As shown in Fig. 2, the mSIS exhibited superior survival predictive ability compared with the 7th AJCC T + N classification in NSCLC patients with BM.

**Fig. 3:**
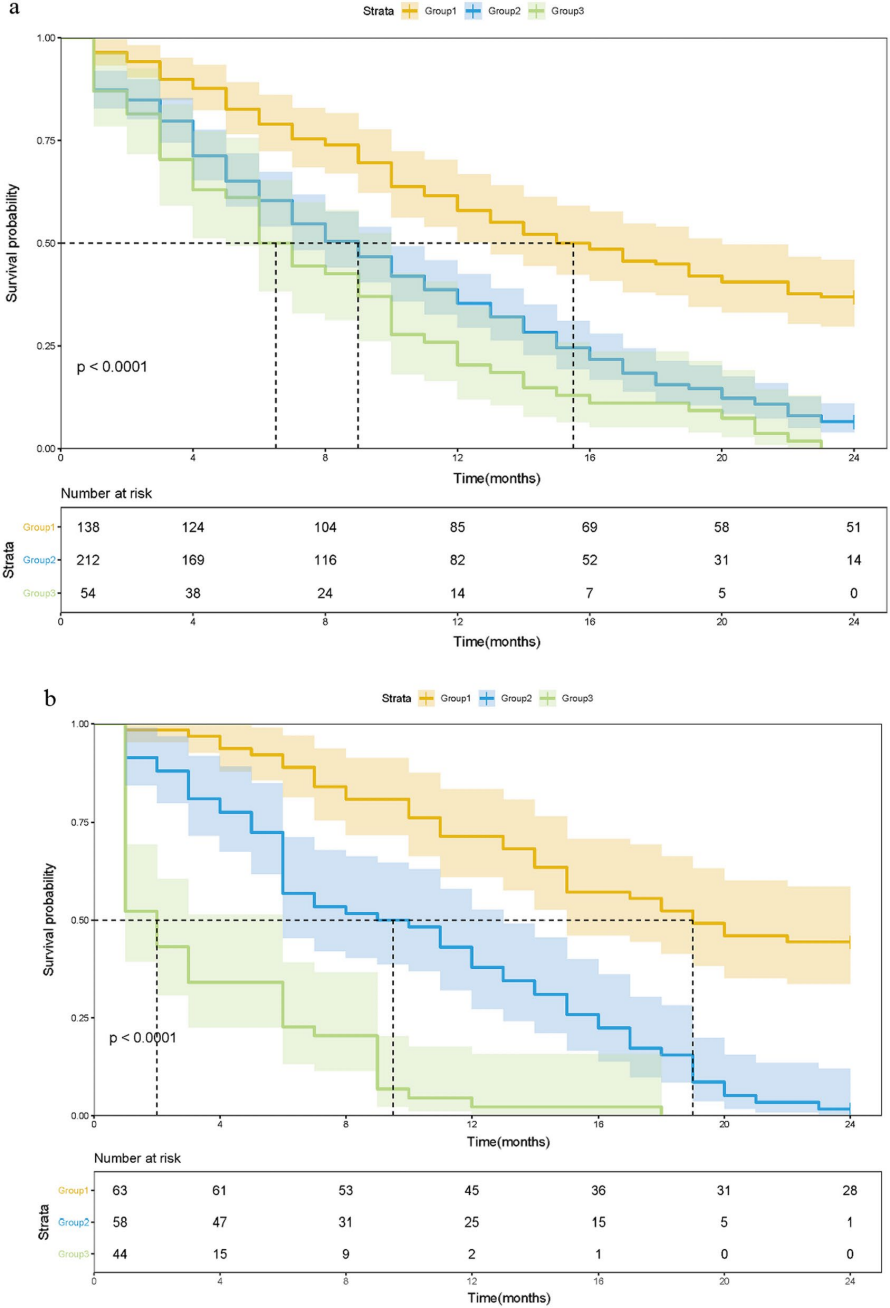
Kaplan–Meier survival analysis for OS according to the mSIS for patients with solitary BM in the training cohort (**a**) and validation cohort (**b**)

**Fig. 4:**
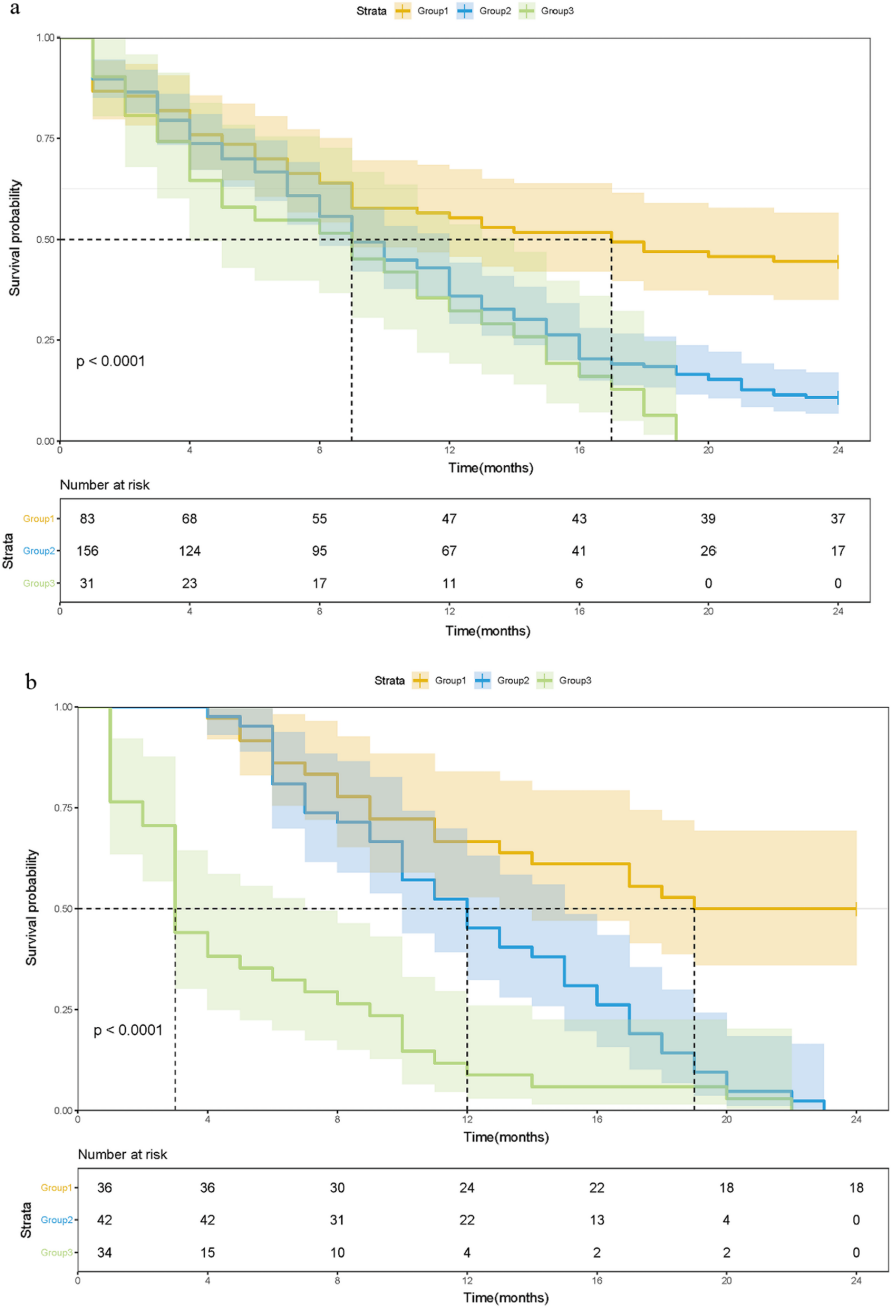
Kaplan–Meier survival analysis for OS according to the mSIS for patients with concurrent extracranial metastasis in the training cohort (**a**) and validation cohort (**b**)

We also investigated the impact of the mSIS in patients with solitary BM or concurrent extracranial metastasis. For patients in the solitary BM subgroup, the 1-year OS rates of these three groups were 61.6%, 38.7% and 25.9%, respectively in the training cohort (P < 0.001, Fig. 3a), and 71.3%, 43.1% and 4.5%, respectively in the validation cohort (P < 0.001, Fig. 3b). For the concurrent extracranial metastasis subgroup of patients, the 1-year OS rates of these three groups were 56.6%, 42.9% and 35.5%, respectively in the training cohort (P < 0.001, Fig. 4a), while there was no significant difference between the medium risk and high-risk group (P = 0.094). In the validation cohort, the 1-year OS rates were 66.7%, 52.4% and 11.8%, respectively (P < 0.001, Fig. 4b). Moreover, in the high risk group, patients less than 40 years old with a history of smoking, the 1-year OS rates were 31.3% and 10.0% in the training and validation cohorts, while the 1-year OS rates of the high-risk group were 29.4% and 7.7%, respectively.

## Discussion

In this study, we summarized the clinical characteristics and pretreatment inflammatory biomarkers of 951 NSCLC patients with BM. The univariate and multivariate analyses suggested that receiving local treatment, or chemotherapy, the NLR and albumin level were independently correlated with prognosis in both the training and validation cohorts. Therefore, we creatively demonstrate the prognostic value of the mSIS (the combination of NLR and albumin level). The mSIS manifested superior survival predictive ability which was also confirmed in ENKTL [14].

There is a strong association between inflammatory cells and tumors. Inflammatory cells and cytokines are more likely to promote tumor growth, progression, and immunosuppression than to produce an effective anti-tumor host response [12]. The relationship between inflammatory response and tumors has become a hot research topic. A large number of studies have suggested that inflammatory cells including neutrophils, lymphocytes, monocytes and acute phase proteins play a significant role in the occurrence, development and prognosis of cancer [15–17]. While albumin is one of the important indicators of malnutrition, it is also closely related to immune incompetence and leads to accelerated tumor progression through the suppression of tumor immunity [18, 19].

To our knowledge, this is the first study to explore the mSIS as a prognostic factor for NSCLC patients with BM based on an unprecedented number of cases. In the training and validation cohorts, the novel prognostic mSIS effectively distinguished between patients with significantly different OS. Patients with a higher mSIS had a worse OS. In the two groups, patients at a low-risk without any adverse factor had 1-year OS rates of 47.0% and 56.0%, patients at medium-risk with one adverse factor had 1-year OS rates of 19.0% and 17.0%, while high-risk patients with two adverse factors had 1-year OS rates of 12.0% and 2.0%, respectively. Thus, it is suggested that low/medium risk patients might receive de-escalation chemotherapy to avoid severe side effects. In addition, in the solitary BM subgroup, the mSIS could also distinguish between the OS rates. The 1-year OS rates of three groups were 61.6%, 38.7% and 25.9%, respectively in the training cohort and 71.3%, 43.1% and 4.5% respectively, in the validation cohort. However, there was no significant difference between the medium risk and high-risk group for the subgroup of patients with concurrent extracranial metastasis in the training cohort. Further research is needed to confirm the underlying difference between solitary BM and concurrent extracranial metastasis. We will expand the sample size to test our results in the future.

Another interesting result was that although young patients smoked, the OS rates were still higher than for elderly patients. We consider that young patients may have better Performance Status (PS) and less underlying diseases, so have more treatment options, and can tolerate more intensive anti-tumor therapy and benefit from it. Younger patients may also have better immune system function, more active neutrophils, and more regenerating T cells. A previous study showed that the lower the level of superoxide dismutase (SOD) in the human body, the less T cell diversity [20].

The peripheral NLR is believed to be a proxy for the ongoing inflammatory process in the tumor microenvironment. A complex body of scientific evidence suggests that neutrophils are associated with pro- and anti-tumor activities in vivo such as enhanced angiogenesis, which contributes to tumor cell proliferation and the potential promotion of metastatic tumor cells [21, 22]. Neutrophils secrete reactive oxygen species and vascular endothelial growth factor (VEGF), which can result in cellular genetic instability, DNA damage and promote the tumor cell cycle by activating the VEGF receptor 2, thereby providing conducive conditions for tumor cell growth [23, 24]. Lymphocytes, on the other hand, are indicators of cell-mediated immunity, and play a central role in the cytotoxic immune response of the host [25]. CD4^+^ T helper type 1 lymphocytes, CD8^+^ cytotoxic T lymphocytes and natural killer T cells are the critical subsets. They not only detect and eliminate pre-malignant and malignant tumor cells directly or indirectly, but also prevent angiogenesis and metastasis [26]. Therefore, an elevated NLR may serve as an independent prognostic factor for OS owing to the counts of increased neutrophils and reduced lymphocytes. Serdar Karakaya et al. reported that a high NLR was correlated with worse pathological complete response rates in locally advanced rectal cancer and emerged as independent predictive marker [27]. Yu Tung Lo et al. found that post-steroid NLR could predict OS in primary central nervous system lymphoma [28]. In our larger sample sized study, an NLR < 4.71 was an independent prognostic factor for decreased OS. This suggests that a lower NLR might be a marker of better prognosis for NSCLC patients with BM.

Serum albumin is considered an indicator of the body’s systemic inflammatory response and nutritional status [29]. A decrease in the serum albumin level may be due to the production of cytokines such as interleukin-6 (IL-6) and IL-1 which may control the production of albumin by hepatocytes [18, 30]. Alternatively, tumor necrosis factor (TNF) increases the permeability of the microvasculature, allowing an increased transcapillary passage of albumin [31]. Therefore, serum albumin has become a general indicator for predicting the survival rate of various cancers. In our investigation, serum albumin < 40 g/L predicted adverse outcomes. However, the current causative relationship between these factors is still questionable, and potential mechanisms need further investigation.

In this study, receiving chemotherapy was an independent prognostic factor for lung cancer patients with BM. Platinum compounds and pemetrexed alone, or in conjunction (etoposide, vinorelbine and so on), are the most commonly used chemotherapeutics against BMs for NSCLC [32]. With the advent of newer chemotherapy drugs, especially molecular targeted drugs such as EGFR-TKIs, ALK-TKIs, the sensitivity of the primary site of BM tumors to chemotherapy appears to be as important as the blood–brain barrier permeability when determining the effect of chemotherapy [8, 9]. Our study shows that the risk of death for patients who received local treatment was significantly reduced. Local treatments including surgery, WBRT, and SRS can effectively relieve symptoms, eliminate brain lesions and have little effect on normal tissue [33–36]. With improvements in medicine, the treatment of NSCLC with BM has ushered in a new era of combined surgical resection, chemotherapy, radiotherapy, targeted therapy and immunotherapy.

Our study has several limitations. As a single-center retrospective study, we were unable to eliminate potential selection bias. It is difficult to maintain heterogeneity in the treatment used for each patient, resulting in different clinical prognosis. Prospective trials are warranted for future study.

In conclusion, our study indicates that the mSIS can be used as an independent prognostic indicator for NSCLC with BM, which is easily obtained through laboratory means and should therefore be included into routine clinical settings. The mechanisms underlying the relationship between the mSIS and survival outcomes in NSCLC patients with BM, and the relationship between tumor microenvironment and immune inflammatory cells needs further exploration.

## Data Availability

All relevant data are within the paper and its Supporting Information files.
